# Association between muscle relaxant dosage and recovery in pediatric otolaryngological surgery: A retrospective cohort study

**DOI:** 10.1371/journal.pone.0346355

**Published:** 2026-04-24

**Authors:** Jingyu Tan, Youtan Liu

**Affiliations:** Anesthesia & Surgery Center, Shenzhen Hospital, Southern Medical University; University of Messina, ITALY

## Abstract

**Background:**

Standardized guidelines for dosing strategies of muscle relaxants—specifically aimed at reducing extubation time and side effects—are currently lacking in pediatric otolaryngological surgery. This retrospective cohort study examined the association between induction dosages of neuromuscular blocking drugs and extubation times in children undergoing ambulatory procedures, aiming to provide evidence for future clinical decision-making.

**Objective:**

This study examined the association between induction doses of muscle relaxants and two key outcomes in pediatric otolaryngological surgery: extubation times and the incidence of neuromuscular blockade-related complications (particularly residual paralysis and its clinical consequences, such as reintubation). The goal was to provide evidence that can inform dosing strategies aimed at accelerating recovery and minimizing adverse events.

**Methods:**

A retrospective review was conducted using electronic medical records of pediatric patients who underwent otolaryngological surgery at Shenzhen Hospital, Southern Medical University;data access for research purposes occurred between June 23, 2020, and June 23, 2021. A total of 90 children, aged 3–12 and classified as ASA I-II, was included in this study. The patients were categorized into three groups based on the muscle relaxant used during surgery: R1 (0.4 mg/kg rocuronium bromide), R2 (0.6 mg/kg rocuronium bromide), and C (0.05 mg/kg cisatracurium). Extracted parameters included mean blood pressure (MBP), heart rate (HR), blood oxygen saturation (SpO₂), Cooper scores, Ramsay Sedation Scale (RSS), Agitation Scale (AS), extubation time, and surgery duration.

**Results:**

The R2 group showed significantly higher mean blood pressure (MBP) than both R1 and C at T1 (both P < 0.05), and higher MBP than R1 and C at T2 (P < 0.01 and P < 0.05, respectively). Heart rate was significantly higher in both rocuronium groups compared to C at T1 and T2 (all P < 0.01). Cooper scores were significantly higher in R2 than in R1 or C (P < 0.01). No significant differences were found in SpO₂ at any time point (including T3 and T4), extubation time, surgery duration, RSS, or AS among groups (P > 0.05). No adverse events were observed in any of the three groups.

**Conclusion:**

Despite the absence of pharmacological reversal, no clinical manifestations of residual neuromuscular blockade—including hypoxemia, reintubation, or severe emergence agitation—were observed in any group.Notably, rocuronium 0.6 mg/kg exhibited rapid onset, superior intubation conditions, and hemodynamic stability; extubation times and emergence quality were comparable among all groups receiving a single intubating dose of 1–1.5 × ED₉₅ of a neuromuscular blocking agent under the same intravenous-inhalation combined anesthesia.

## 1. Introduction

Neuromuscular blocking agents (NMBAs) are essential in pediatric anesthesia for tracheal intubation and optimizing surgical conditions. However, their use presents a clinical dilemma, particularly in pediatric otorhinolaryngology (ORL) surgery. While NMBAs facilitate secure airway management, they carry the well-documented risk of postoperative residual neuromuscular blockade (PRNB). PRNB significantly contributes to respiratory morbidity in the post-anesthesia care unit (PACU), including hypoxemia and upper airway obstruction, and may occur even after a single intubating dose [1,2].

This risk is heightened in children undergoing short-duration procedures such as adenotonsillectomy, which is the primary surgical treatment for pediatric obstructive sleep apnea-hypopnea syndrome (OSAHS) [3]. This population represents a unique “double-hit” scenario. First, both the surgery and OSAHS are independently associated with a higher baseline incidence of perioperative respiratory adverse events (PRAEs) [3–5]. Second, the pharmacokinetics and pharmacodynamics of NMBAs in children vary significantly across the pediatric age spectrum, rendering extrapolation from adult data unreliable [6,7]. A recent systematic review by Vanlinthout et al. [8] highlighted this variability, demonstrating that spontaneous recovery times after a single NMBA dose differ considerably from neonates to adolescents.

In response to the risks of PRNB, expert guidelines advocate for quantitative neuromuscular monitoring and proactive reversal as the gold standard [1,2,9]. However, a considerable gap remains between this ideal and routine clinical practice, particularly in ambulatory pediatric surgery. For brief procedures like adenotonsillectomy, NMBAs are used primarily for intubation, and profound intraoperative relaxation is often unnecessary. Consequently, a “simplified pathway” relying on clinical assessment and natural metabolism is common, driven by practical constraints like limited monitor availability and pressures for high operating room turnover [10,11].

Critically, within this widespread simplified pathway, the choice of NMBA and its induction dose lacks robust, procedure-specific evidence. Selection often varies based on individual experience and habit rather than data [12]. While general dosing ranges (e.g., 1–1.5 ED₉₅) are known, there is a striking paucity of research directly comparing the clinical outcomes of different standard intubating doses of short-acting NMBAs specifically in children undergoing adenotonsillectomy. This leaves a critical evidence gap: does the choice between, for example, 1.0 × vs. 1.5 × ED₉₅ of rocuronium meaningfully influence key practical outcomes—such as time to extubation, PACU readiness, or the risk of early respiratory events—in this vulnerable group?This retrospective study leverages real-world clinical data to address this gap by analyzing the association between commonly used NMBA dosing regimens (1–1.5ED₉₅ of rocuronium or cisatracurium) and key perioperative outcomes. By examining intubation conditions, hemodynamic stability, recovery times, and emergence quality, this study aims to provide pragmatic, context-specific evidence to refine anesthesia management and guide clinical decision-making for this large and distinct pediatric surgical population.

## 2 Materials and methods

### 2.1. Study design and population

This retrospective review included data from 90 children aged 3–12 years, classified as ASA physical status I–II, who underwent elective adenotonsillectomy (n = 38) or adenoidectomy alone (n = 52) between June 23, 2020, and June 23, 2021. Data were accessed for research purposes during the same period.Due to variations in clinical practice among senior resident anesthesiologists, children received different muscle relaxants and dosages and were accordingly divided into the following groups: R1 group (rocuronium bromide 0.4 mg/kg), R2 group (rocuronium bromide 0.6 mg/kg), and C group (cisatracurium 0.05 mg/kg). Complete medical records and follow-up information were obtained for the study, which was approved by the Medical Ethics Committee of Shenzhen Hospital of Southern Medical University (EC No. NYSZYYEC20200003). The need for informed consent was waived by the ethics committee because this retrospective study used fully anonymized medical records. All patient identifiers were removed prior to data access and analysis to ensure confidentiality. This retrospective study utilized existing clinical data; anesthesia protocols were determined solely by clinical teams without research intervention. Group assignments (R1/R2/C) were defined post hoc based on administered doses.

**Exclusion criteria:** Preoperatively assessed difficult airway (including previous difficult intubation history);

Known allergy to neuromuscular blocking agents;

Severe obstructive sleep apnea (OSA, AHI > 30);

Chronic kidney disease (CKD stage ≥3);

Congenital airway anomalies or neuromuscular disorders;

Acute/chronic respiratory tract infections;

Neuroleptic drug allergies;

Mental disorders or cognitive impairment;

Congenital heart disease;

Neurological system dysfunctions;

Administration of non-protocol neuromuscular blocker doses.

### 2.2. Study methods

#### 2.2.1. Preoperative preparation and anesthetic induction.

Patients were instructed to fast from solid foods for 6–8 hours and from liquids for 2–3 hours before the surgical procedure. In the ward, venous access was secured in the upper limb, and 250 ml of a 5% glucose saline solution was administered, adhering to the 4-2-1 protocol. The induction process followed institutional standardized protocols (SOPs), and all anesthesia procedures were performed by clinical teams. This study retrospectively analyzed data from medical records without research intervention. Prior to induction, children received preoxygenation with 100% oxygen via facemask (typically 6–8 L/min for 3 minutes when clinically feasible). The following sequence of intravenous medications was administered: dexmedetomidine at 0.3 μg/kg, sufentanil at 0.3 μg/kg, lidocaine at 1 mg/kg, propofol at 2.5 mg/kg, a neuromuscular blocker, and dexamethasone at 0.2 mg/kg. Anesthesia induction was achieved solely with intravenous agents as described. Sevoflurane was not used during induction. After intravenous propofol administration (2.5 mg/kg), loss of eyelash reflex and consciousness was confirmed. Immediately following this, mask ventilation was initiated to assess airway patency by observing chest rise, capnography waveform (PₑₜCO₂), and stable SpO₂ (≥98%). Neuromuscular blocking agents were administered only after confirming unobstructed mask ventilation. Patients were allocated to one of three groups based on the neuromuscular blockers used: the R1 group received rocuronium bromide at 0.4 mg/kg (1x ED₉₅), the R2 group (1.5x ED₉₅) received rocuronium bromide at 0.6 mg/kg, and the C group received cisatracurium at 0.05 mg/kg (1x ED₉₅). Tracheal intubation was timed according to the recommended onset of neuromuscular blockade per Chinese neuromuscular blocking agent guidelines (4 minutes, 90 seconds, and 5 minutes [9] for each respective group), with intubation conditions retrospectively analyzed using Cooper scores documented in anesthesia records.

#### 2.2.2. Anesthetic maintenance and ventilation.

Following successful intubation, the endotracheal tube was connected to a Drager anesthesia machine for intermittent positive pressure ventilation, with a tidal volume of 8–10 ml/kg and a respiratory rate of 18–24 breaths per minute. Anesthesia was maintained with 2–3 Vol% sevoflurane at a total fresh gas flow of 2 L/min, with a fraction o f inspired oxygen of 0.5. Anesthesia depth was maintained via continuous intravenous infusion of remifentanil at 0.2–0.3 μg/kg/min to ensure hemodynamic stability throughout the procedure. Intraoperative monitoring included blood pressure (BP), heart rate (HR), blood oxygen saturation (SpO₂), partial pressure of end-tidal carbon dioxide (PₑₜCO₂), MAC, and electrocardiogram.

#### 2.2.3. Postoperative management.

At the conclusion of surgery, sevoflurane administration was ceased, and high-flow fresh air was supplied to expel inhaled anesthetics. Postoperative analgesia was managed with an intravenous infusion of tramadol at 0.2 mg/kg. Any instances requiring muscle-relaxant antagonists were recorded. Continuous SpO₂, MAP, PₑₜCO₂ and ECG monitoring were maintained in the Post-Anesthesia Care Unit (PACU); forehead temperature was documented at arrival and departure. The anesthesiologist, blinded to the specific muscle relaxant and dosage administered, evaluated the child’s recovery and determined extubation timing based on standardized criteria: regular spontaneous breathing (respiratory rate 15–30 breaths/min), adequate tidal volume (>8 ml/kg), response to commands (e.g., head raising test >5 sec, swallowing reflex). Children were extubated upon regaining consciousness (eye-opening or purposeful movement) and meeting these criteria. Pre-extubation checks included a cuff leak test, immediate availability of reintubation equipment, thorough oropharyngeal suctioning, and lateral positioning. Post-extubation, all patients received immediate mask oxygen therapy (FiO₂ 40%−50% at 5–8 L/min) for 3–5 minutes, transitioning to nasal cannula (1–2 L/min) if SpO₂ ≥ 95% on room air (FiO₂ 0.21) with stable respiration (RR < 30 breaths/min). Patients were subsequently monitored in PACU for 0.5–1 hour.

### 2.3. Data collection and measurement

Hemodynamic parameters, including mean arterial pressure (MAP), heart rate (HR), and peripheral oxygen saturation (SpO₂), were recorded at five predefined time points: T0: immediately before tracheal intubation (baseline); T1: 1 minute after intubation; T2: 5 minutes after intubation; T3: 1 minute after extubation; T4: 5 minutes after extubation.

Emergence quality was assessed at the corresponding time point after extubation: Ramsay Sedation Scale (RSS), a 6-point scale ranging from 1 (anxious/agitated) to 6 (unresponsive); 5-point Agitation Scale (AS), ranging from 1 (calm) to 5 (severely agitated with risk of harm).

All scores were documented in the anesthesia records by attending anesthesiologists who were unaware of the group assignment.

Surgery duration was defined as the interval from confirmed complete hemostasis in the bilateral tonsillar fossae and adenoid surgical field (following complete resection of target tissues) to endoscope withdrawal from the oral cavity, with stable vital signs in children.

Extubation time was defined as the interval from discontinuation of sevoflurane administration to actual removal of the endotracheal tube.

### 2.4. Tracheal intubation assessment and scores

This study utilized the Cooper scoring method to evaluate the conditions during tracheal intubation [13]. Because it reflects optimal intubating conditions, achieving a ‘good’ or ‘excellent’ Cooper score is associated with a lower risk of airway trauma and respiratory complications (such as laryngospasm or bronchospasm). The assessment criteria included the difficulty of laryngoscopy, the condition of the vocal cords, and the patient’s response to intubation, such as coughing. The scoring system uses a scale from 0 to 9, with the following interpretations:a score of 8–9 indicates excellent intubation conditions, 6–7 indicates good conditions, 3–5 indicates fair conditions, and 0–2 indicates poor conditions. In clinical practice, scores rated as ‘good’ or ‘excellent’ are considered acceptable. In accordance with routine clinical practice at our institution, all tracheal intubations and the subsequent clinical assessments of intubation conditions (as documented via the Cooper score in the anesthesia records) were performed jointly by the same senior associate chief physician (the corresponding author) and a senior resident. This consistent clinical pairing was standard care during the study period.

### 2.5. Outcomes

#### 2.5.1. Primary outcome.

The primary outcome of this study was extubation time, defined as the time interval from discontinuation of sevoflurane administration to the actual removal of the endotracheal tube, assessed by attending anesthesiologists blinded to group assignment based on standardized clinical criteria (regular spontaneous breathing, tidal volume >8 mL/kg, eye opening or response to commands, and recovery of swallowing reflex).

#### 2.5.2. Secondary outcomes.

Tracheal intubation conditions — assessed by Cooper score (see Section 2.4).

Hemodynamic stability — MAP, HR, and SpO₂ at T0-T4 (including T3 and T4, see Section 2.3).

Emergence quality — RSS and AS scores immediately post-extubation (see Section 2.3).

Surgery duration — as defined in Section 2.3.

Incidence of adverse events — all events were predefined according to the Chinese Expert Consensus on Definitions of Perioperative Adverse Events (2021) [14], and included:

Respiratory: reintubation, laryngospasm, or SpO₂ < 90% for >60 seconds;

Cardiovascular: Hemodynamic instability: MAP change >30% from baseline for >5 consecutive minutes; Bradycardia: HR < 60 bpm; Hypotension: MAP < 55 mmHg (age 3 years) or <60 mmHg (age 4–12 years);

Hypothermia: skin temperature <36°C;

Agitation requiring pharmacological intervention (e.g., dexmedetomidine).

### 2.6. Statistical analysis

Statistical analysis was performed using SPSS version 26.0 software (IBM Corp., Armonk, NY, USA).This study is a retrospective observational study. Due to the limitations of the clinical data collection scope, it is difficult to conduct strict sample size estimation as in a prospective clinical trial. To minimize the risk of type II error (false negative), this study has adopted multiple quality control measures.

#### 2.6.1. Data description.

Normality of continuous variables was assessed using the Shapiro–Wilk test. Normally distributed data are presented as mean ± standard deviation (SD), while non-normally distributed data are expressed as median with interquartile range [M (IQR)]. Categorical variables are reported as frequencies and percentages [n (%)].

**Baseline characteristics:** Normality was assessed using the Shapiro–Wilk test, and homogeneity of variances using Levene’s test. For normally distributed continuous variables with equal variances, one-way ANOVA was used; if variances were unequal, Welch’s ANOVA was applied. Non-normally distributed continuous variables were analyzed using the Kruskal–Wallis H test. Categorical variables were compared using the chi-square test or Fisher’s exact test, as appropriate.

**Primary outcome (extubation time):** Due to violation of normality in some groups (Shapiro–Wilk test, P < 0.05), the Kruskal–Wallis H test was employed for overall comparison among the three groups. If significant, post-hoc pairwise comparisons were performed using Dunn’s test with Bonferroni correction (adjusted significance threshold: α = 0.017). Surgery duration, though a secondary outcome, was analyzed using the same approach due to similar distributional characteristics.

#### 2.6.2. Secondary outcomes.

Ordinal variables (Cooper score, Ramsay Sedation Scale, Agitation Scale) were analyzed using the Kruskal–Wallis H test, followed by Dunn–Bonferroni post-hoc tests if overall significance was reached.

Hemodynamic parameters (MAP, HR, SpO₂) at each time point were compared between groups using the same methods as for baseline data: for normally distributed data with equal variances, one-way ANOVA followed by Tukey’s HSD post-hoc tests was used; for data with unequal variances, Welch’s ANOVA with Games–Howell post-hoc tests was applied; for non-normally distributed data, the Kruskal–Wallis H test was used.

Adverse events were compared using the chi-square test or Fisher’s exact test. Due to the low event rate, results were primarily descriptive, and no multiplicity adjustment was applied.

#### 2.6.3. Significance level, multiplicity adjustment, and exploratory nature.

All statistical tests were two-tailed. For overall comparisons (e.g., ANOVA, Kruskal–Wallis H test), a P value < 0.05 was considered statistically significant. For post-hoc pairwise comparisons, the Bonferroni correction was applied to control the family-wise error rate, with an adjusted significance threshold of α = 0.017 (0.05/3) for comparisons among the three groups.

Given the retrospective and exploratory nature of this study, no subgroup analyses were pre-specified. To mitigate the risk of type I error inflation due to multiple testing, results of post-hoc analyses are reported primarily as effect estimates with 95% confidence intervals (CIs), with P values provided as descriptive references rather than confirmatory evidence. All findings should be interpreted as hypothesis-generating and warrant validation in prospective studies.

#### 2.6.4. Effect size estimation.

To enhance clinical interpretability, between-group differences are reported as mean differences with 95% confidence intervals (CIs) for normally distributed variables, and as median differences with 95% CIs (estimated using the Hodges–Lehmann method) for non-normally distributed variables.

## 3. Results

### 3.1. Comparison of general data

The gender, age and BMI of the three groups of children all showed non-normal distribution after Shapiro–Wilk test (all P < 0.05), so the Kruskal–Wallis H test was used for comparison among the groups Table 1.

**Table 1:** Comparison of general data.

Groups	n	Gender (case, M/F)	Ages (y) [M (IQR)]	BMI (kg/m²) [M (IQR)]
R1	30	20/10	4.5 (3)	15.65 (2.86)
R2	30	17/13	6 (4)	15.00 (2.77)
C	30	19/11	5 (3)	15.40 (2.02)
**p-value**	–	0.718	0.101	0.387

Statistical analysis showed that there were no statistically significant differences in gender, age and BMI among the three groups (all P > 0.05), suggesting that the baseline data of the three groups were well balanced and comparable. This excluded the interference of the aforementioned confounding factors on the subsequent observation indicators, thereby enhancing the reliability of the group comparisons and the credibility of the results.

### 3.2. Comparison of MAP, SpO₂ and HR obtained in the three groups of children during anesthesia

For comparison among the three groups at all time points (i.e., T0-T4), MAP and HR showed statistically significant differences at T1 and T2 (P < 0.05), while MAP, SpO₂ and HR showed no statistically significant differences at time points T0, T3 and T4 (P > 0.05). (Table 2)

**Table 2:** Comparison of perioperative vital signs among the three groups.

Groups	Time point	MBP/mmHg	SpO₂/%[M（IQR）]	HR/bpm
R1	T0	79.3 ± 10.0	100 (1)	95.2 ± 13.9
	T1	72.3 ± 11.8*^c^	100 (0)	98.1 ± 15.5**^f^
	T2	64.5 ± 9.5**^d^	100 (0)	88.3 ± 13.0**^f^
	T3	87.8 ± 11.4	98 (2)	103.2 ± 13.3
	T4	82.0 ± 11.9	98 (2)	101.0 ± 11.8
R2	T0	82.1 ± 10.9	100 (1)	90.4 ± 16.5
	T1	80.7 ± 12.0*^a^	100 (0)	93.7 ± 12.5**^f^
	T2	72.4 ± 9.8**^bf^	100 (0)	87.1 ± 12.3**^f^
	T3	88.7 ± 11.9	98 (3)	99.3 ± 16.2
	T4	83.1 ± 8.6	98 (1)	99.4 ± 13.5
C	T0	80.7 ± 10.4	99 (1)	90.2 ± 15.4
	T1	71.5 ± 9.0**^d^	100 (0)	81.3 ± 12.0**^bd^
	T2	62.3 ± 9.0**^d^	100 (0)	76.1 ± 13.0**^bd^
	T3	90.9 ± 11.1	98 (2)	98.3 ± 20.4
	T4	86.2 ± 12.0	98 (2)	97.1 ± 15.3

Note: * indicates a statistically significant inter-group difference (P < 0.01). **indicates an inter-group difference (P < 0.05). At the same time point, compared with R1 group, ᵃP < 0.05, ᵇP < 0.01; Compared with R2 group, ᶜP < 0.05, ᵈP < 0.01; Compared with C group, ᵉP < 0.05, ᶠP < 0.01.The SpO_2_ values show a non-normal distribution, which is represented by the median (IQR).

At T1, MBP in the R2 group was higher than in R1 group (P = 0.022 < 0.05, Mean difference 8.4, 95% CI: [−15.79, −1.01]) and C group (P = 0.04 < 0.05, Mean difference 9.23, 95% CI: [2.63, 15.84]).

There was no difference between the R1 and R2 groups (P > 0.05) in HR, but the difference between R1-C, R2-C was more significant (R1-C, P = 0.000 < 0.01, Mean difference 16.917, 95% CI: [8.41, 25.52]; R2-C, P = 0.001 < 0.01, Mean difference 12.433, 95% CI: [−20.03, −4.84]), indicating that the heart rate of R groups was faster than C group (P < 0.01) at T1. See Table 2.

At T2, there was no difference between the R1 and C groups (P > 0.05) in MBP, while the difference between R2-R1(P = 0.004 < 0.01, Mean difference 8.333, 95% CI: [2.34, 14.32]), R2-C groups (P = 0.000 < 0.01, Mean difference 10.167, 95% CI: [4.35, 15.99]) was more significant, indicating that the blood pressure of the R2 group was higher than that in other groups (P < 0.01). There were no differences between the R1 and R2 groups (P > 0.05) in HR, but the differences between R1-C, R2-C were more significant (R1-C, P = 0.001 < 0.01, Mean difference 12.833, 95% CI: [4.53, 21.14]; R2-C, P = 0.004 < 0.01, Mean difference 11.067, 95% CI: [3.19, 18.94]), indicating that the heart rate of R groups was faster than that of Group C at T2.

From 1 to 5 minutes after induction, the decreasing trend in blood pressure and heart rate was more pronounced in Group C than in Groups R. From 1 to 5 minutes after induction, the decreasing trend in blood pressure and heart rate was more pronounced in Group C than in Groups R. There were no significant differences in vital signs between the groups 1 or 5 min after extubation Table 3.

**Table 3:** Significant between-group differences (Mean difference [95% CI]).

Time	Comparison	Parameter	Mean difference [95% CI]	p-value*
T1	R1 vs R2	MBP	−8.400 [−15.79, −1.01]	0.022*
	R1 vs C	MBP	0.833 [−5.70, 7.37]	0.949
	R2 vs C	MBP	9.233* [2.63, 15.84]	0.04*
	R1 vs R2	HR	4.533 [−4.14, 13.21]	0.425
	R1 vs C	HR	16.917* [8.41, 25.52]	0.000**
	R2 vs C	HR	12.433* [−20.03, −4.84]	0.001**
T2	R1 vs R2	MBP	−8.333* [−14.32, −2.34]	0.004**
	R1 vs C	MBP	1.833 [−3.92, 7.59]	0.725
	R2 vs C	MBP	10.167* [4.35, 15.99]	0.000**
	R1 vs R2	HR	1.767 [−6.34, 9.87]	0.860
	R1 vs C	HR	12.833* [4.53, 21.14]	0.001**
	R2 vs C	HR	11.067* [3.19, 18.94]	0.004**

Tukey’s HSD adjusted p-values, *P < 0.05, **P < 0.01

### 3.3. Comparison of tracheal intubation scores among the three groups

**Fig 1:**
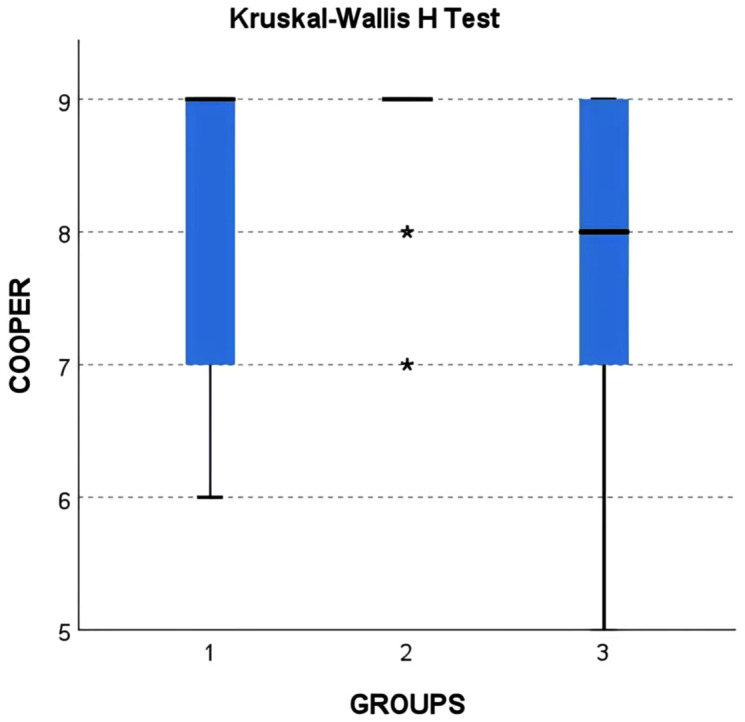
Comparison of Cooper score.

Postintubation Cooper scores differed significantly among groups R1, R2, and C (P < 0.01, Fig 1, Table 4). Overall, most patients in all groups achieved excellent intubation conditions, confirming clinically satisfactory tracheal intubation. The Cooper score was slightly lower in groups R1 and C than in group R2, with some patients in R1 and C rated as good, which remained clinically acceptable.

**Table 4:** Comparison of each component of Cooper score.

Group	Cooper score[M（IQR）]	each component of Cooper score		
		Laryngoscopy[M（IQR）]	condition of the vocal cords[M（IQR）]	Intubation response[M（IQR）]
R1	9 (2)	3 (0)	3 (0)	3 (1)
R2	9 (0)	3 (0)	3 (0)	3 (0)
C	8 (2)**	3 (0)	3 (0)	2.5(2)**
**P-value**	0.011*	0.602	0.451	0.004**

*P < 0.05 for overall comparison; **P < 0.01 vs. R2 group in post-hoc analysis (Bonferroni-corrected).

As shown in Table 4, there were no significant intergroup differences in laryngoscopic view or vocal cord conditions (all P > 0.05). In contrast, intubation response differed significantly among the three groups (P = 0.011 < 0.05). Post-hoc pairwise comparisons (Table 5) revealed a significant difference in intubation response only between R2 and C (mean difference 0.700, 95% CI 0.25–1.15, adjusted P = 0.003 < 0.01), with no significant differences between R1 vs. R2 or R1 vs. C.

**Table 5:** Post-hoc pairwise comparisons of Cooper score (Intubation response,Kruskal–Wallis H+Dunnett+Bonferroni correction).

Comparison	Mean difference	95% CI	Adjusted P-value
R1&R2	−0.433	−0.83,-0.04	0.115
R1&C	0.267	−0.28,0.82	0.701
R2&C	0.700	0.25,1.15	0.003**

p-values *P < 0.05,**P < 0.01.

These findings indicate that the 1.5 × ED₉₅ dose (R2) provided superior intubation response compared with the 1 × ED₉₅ dose (C). The similar results between R1 and C suggest comparable intubation conditions at the same 1 × ED₉₅ dose level. The lack of a significant difference between R2 and R1 may be attributed to the limited sample size and the conservative nature of the Kruskal–Wallis nonparametric test.

In summary, 1 × ED₉₅ is sufficient for satisfactory clinical intubation, while 1.5 × ED₉₅ can further improve intubation conditions, primarily by reducing intubation response (Fig 1; Tables 4 and 5).

### 3.4. Comparison of Surgery and Extubation Time Among the Three Groups

No statistically significant differences were observed among the three groups (R1, R2, and C) in surgery duration or time to extubation after discontinuation of anesthesia (P > 0.05). This suggests that the choice of different neuromuscular blockers and dosages did not significantly impact the duration of surgery or the time taken to remove the endotracheal tube after the surgery. (Table 6)

**Table 6:** Comparison of extubation and Surgery time among the three groups.

Group	N	Extubation time/min[M（IQR）]	Surgery time/min[M（IQR）]
R1	30	21.5 (18.5-27.0)	26.5 (19.0-32.50)
R2	30	21.0 (15.8-25)	29.0 (20.0-38.0)
C	30	23.0 (18.8-28)	24.0 (17.0-29.0)
p-value	–	0.518	0.111

### 3.5. Comparison of emergence quality (RSS and AS) among the three groups

No statistically significant differences were observed in Ramsay Sedation Scale (RSS) or Agitation Scale (AS) scores among the three groups immediately after extubation (P > 0.05 for all comparisons). These findings indicate that the choice and dosage of neuromuscular blocking agents did not influence early emergence quality in this pediatric cohort (Table 7).It is well recognized that emergence agitation may be associated with inadequate sedation and analgesia, as well as with hypoxemia. In the present study, the absence of significant differences in AS scores among groups, coupled with the stable SpO₂ levels observed throughout the perioperative period (as shown in Table 2), suggests that the observed low agitation levels were unlikely to be confounded by these factors.

**Table 7:** Comparison of emergence quality among the three groups[M (IQR)].

Group	n	T3 Ramsay Score	T4Ramsay Score	T3 Agitation Score	T4Agitation Score
R1	30	3(2)	3(2)	1(1)	1(1)
R2	30	2(2.25)	2(2)	1(1)	1(1)
C	30	3(2)	3(1)	1(0.25)	1(0)
P-value	–	0.759	0.770	0.464	0.138

^P^ > 0.05 for all intergroup comparisons by Kruskal-Wallis H test

### 3.6. Intraoperative fluid management and blood loss

All patients received 250–500 ml of crystalloid solution during the procedure, based on weight and hemodynamic status. Estimated blood loss ranged from 5 to 10 mL in all cases, with no transfusions or hemodynamic intervention required.

### 3.7. Adverse events

No adverse events occurred in any group. No additional muscle relaxants were required intraoperatively in any group (R1, R2, C), and no patients required reversal agents for neuromuscular blockade. This suggests that the chosen agents and dosages were adequate for maintaining the necessary blockade throughout surgery and did not produce residual effects that would impair postoperative respiration.

## 4. Discussion

### 4.1. Rationale for muscle relaxant use in pediatric otolaryngological surgery

Although some institutions avoid muscle relaxants during pediatric adenotonsillectomy, evidence suggests that high-concentration sevoflurane without NMBAs increases the risk of myocardial depression, hypotension [15], agitation [16,17], and seizure-like EEG activity [18,19] in children. Consequently, expert guidelines [20,21] recommend the use of muscle relaxants to facilitate endotracheal intubation, optimize surgical conditions, and minimize airway trauma.

### 4.2. Pharmacological profiles of rocuronium and cisatracurium

Rocuronium and cisatracurium are the most commonly used intermediate-acting NMBAs in pediatric ENT surgery [22–25]. However, research on 1-2x ED₉₅ dosing regimens remains limited. In this study, we relied on established research to determine the 1x ED₉₅ dosages of rocuronium bromide and cisatracurium, as well as their expected onset times, for use in pediatric otolaryngological surgery [26,27]. Our study aimed to evaluate the association between different muscle relaxant doses and both surgical condition adequacy and ambulatory procedural efficiency.

Rocuronium, an aminosteroid NMBA, is valued for its rapid onset and hemodynamic stability [27]. Although 0.6 mg/kg (1.5 × ED₉₅) is lower than the 2 × ED₉₅ dose (0.8 mg/kg), sometimes suggested for children, it achieved satisfactory intubation conditions within 90 seconds [15,28], likely due to children’s higher cardiac output accelerating drug distribution. In our cohort, the R2 group exhibited faster heart rates at T1 and T2 compared to the C group, a physiologically relevant advantage given children’s heart rate-dependent cardiac output. Moreover, R2 demonstrated the smallest post-induction decrease in blood pressure—likely attributable to earlier intubation, preserved sympathetic tone, and reduced cumulative circulatory suppression from induction agents.

Cisatracurium, a benzylisoquinoline NMBA, offers organ-independent metabolism and cardiovascular stability [28,29]. However, its 5-minute onset time at 1 × ED₉₅ may prolong the intubation waiting period, potentially affecting workflow efficiency. The more pronounced hypotension and bradycardia observed in group C likely reflect the combined circulatory effects of propofol, sufentanil, and delayed airway instrumentation.

### 4.3. Impact of sevoflurane on neuromuscular blockade and extubation time

Sevoflurane maintenance exerts synergistic effects with NMBAs, prolonging their duration of action and reducing dose requirements [30]. In our study, extubation times did not differ significantly among groups (P > 0.05), consistent with findings by Huh et al. [17] who reported no extubation delay despite varying rocuronium doses under sevoflurane anesthesia. Conversely, Bartolek et al. [31], who avoided volatile agents entirely, observed longer recovery to T90 for both 0.45 mg/kg (27.2 ± 0.86 min) and 0.6 mg/kg rocuronium (36.3 ± 0.53 min). These contrasting results underscore the significant impact of sevoflurane on neuromuscular recovery kinetics.

Notably, no patient in our cohort received reversal agents, yet all met rigorous extubation criteria without clinical evidence of residual weakness. Huh et al. [17] similarly found that neostigmine administration did not shorten extubation time under sevoflurane maintenance. Furthermore, Kim et al. [32] demonstrated that sevoflurane may delay sugammadex-mediated reversal by modulating A1 receptors at the neuromuscular junction. Collectively, these findings suggest that when sevoflurane is used for maintenance, even single-dose NMBAs do not significantly prolong extubation, and the routine use of reversal agents may offer limited benefit in this specific context.

Although previous studies have demonstrated that sevoflurane significantly prolongs the duration of intermediate-acting neuromuscular blocking agents (NMBAs) (by approximately 50%), the clinical relevance of this prolongation must be interpreted within the context of the specific patient population and dosage strategy employed. Previous research has established a log-linear relationship between NMBA dose and duration of action [7], suggesting that the temporal variability introduced by drug interactions can be actively managed through precise dose titration. This concept is supported by our clinical observations in children aged 3–12 years. In this cohort, the use of a low-dose NMBA combined with sevoflurane maintenance resulted in a duration of neuromuscular block that was well-suited to the surgical procedure, with spontaneous recovery to a safe extubation threshold (TOFr ≥ 0.9) by the end of surgery. Notably, this approach was not associated with an increase in postoperative respiratory adverse events. This retrospective finding suggests that, in a specific pediatric age group and for select surgical procedures, the combination of a low-dose NMBA and sevoflurane may reside within an optimal “therapeutic window”—balancing effective surgical relaxation with a clinically acceptable, and spontaneously reversible, degree of prolongation.

### 4.4. Clinical implications of rocuronium 0.6 mg/kg Dosing

While expert guidelines recommend age-stratified NMBA dosing [20], our data indicate that for brief pediatric otolaryngological surgeries (e.g., adenotonsillectomy) under sevoflurane anesthesia, a single 0.6 mg/kg rocuronium dose, though lower than the 2 × ED₉₅ dose (0.8 mg/kg) suggested for children, achieved excellent intubation conditions (Cooper score 9(0)), stable hemodynamics (MBP 80.7 ± 12.0 mmHg at T1), and no prolongation of extubation time (21.0 (15.8–25) min). These findings support its pragmatic utility in ambulatory settings where rapid turnover and safety are paramount.

### 4.5. Clinical safety: absence of hypoxemia and reintubation

From a patient safety perspective, the most critical finding of this study is the absence of hypoxemia or unplanned reintubation across all groups. Although Vanlinthout et al. [7] described inter-individual and age-related variability in spontaneous recovery from neuromuscular blocking agents—which theoretically increases the risk of postoperative residual neuromuscular blockade (PRNB)—all patients in this cohort completed the perioperative period without adverse events, despite the absence of quantitative monitoring, by strictly adhering to standardized clinical extubation criteria (e.g., consciousness, response to commands, adequate tidal volume). This suggests that, in this carefully selected low-risk pediatric population, systematic clinical assessment combined with an understanding of the pharmacokinetic/pharmacodynamic profile of a single intubating dose may be sufficient to prevent immediate safety events related to PRNB.

The comparable recovery outcomes across groups further support that, when selecting 1–1.5 × ED₉₅ doses, anesthesiologists can prioritize optimizing intubation conditions and maintaining hemodynamic stability without compromising recovery safety. These findings provide context-specific empirical support for the pharmacological principles underpinning recent neuromuscular management guidelines [9,7,33–35]—namely, that spontaneous recovery following a single intubating dose is predictable within a clinically relevant timeframe in this age group.

It must be acknowledged, however, that our study lacks quantitative neuromuscular monitoring (train-of-four ratio), which is now strongly recommended by guidelines to minimize PRNB risk. While we observed no overt adverse events, the absence of monitoring precludes definitive conclusions about the incidence of subclinical residual blockade, which has been associated with postoperative pulmonary complications. This limitation is inherent to our retrospective design and reflects real-world practice during the study period (2020–2021), prior to the widespread adoption of recent monitoring guidelines.

Importantly, our findings do not challenge the value of quantitative monitoring but rather highlight a complementary perspective. In low-risk children meeting our strict inclusion criteria, a simplified approach combining a single 1–1.5 × ED₉₅ dose with rigorous clinical assessment ensured immediate safety. This raises the question of whether, in this specific context, the incremental benefit of routine monitoring justifies its routine use—a hypothesis that warrants prospective investigation. Future studies incorporating quantitative monitoring are needed to determine whether, and in which populations, monitoring-driven protocols improve outcomes beyond those achievable with rigorous clinical assessment alone.

### 4.6. Study limitations

This study has several limitations that warrant discussion.

First, the single-center retrospective design and relatively modest sample size (N = 90, 30 per group) may limit the generalizability of our findings and reduce statistical power to detect differences in low-frequency adverse events. Additionally, despite efforts to control for confounders, the potential for selection bias cannot be fully excluded. These limitations highlight the need for larger, multicenter prospective studies with objective neuromuscular monitoring to validate our observations and establish definitive dosing guidelines.

Second, the absence of quantitative neuromuscular monitoring (e.g., train-of-four ratio) is a significant methodological limitation. Without objective data, the exact depth of blockade and the precise moment of full neuromuscular recovery cannot be definitively confirmed for each patient.

However, the recent systematic review by Vanlinthout et al. [7] provides important context for interpreting this limitation. Their meta-analysis demonstrated that following a single intubating dose of rocuronium or cisatracurium, spontaneous recovery to a TOF ratio ≥0.9 in children aged 2–12 years consistently occurs within a predictable timeframe—typically well within the duration of short ENT procedures, even without reversal agents. This pharmacodynamic evidence suggests that, in the specific context of single-dose administration for brief surgeries, the risk of clinically significant residual paralysis at the time of extubation may be inherently low.

Our clinical findings align with this pharmacological rationale. Despite the absence of monitoring, no patient in any group exhibited clinical signs of residual blockade, and there were no cases of hypoxemia or reintubation. This suggests that, when combined with rigorous, standardized clinical extubation criteria (regular breathing, tidal volume >8 mL/kg, eye opening, intact swallowing), the predictable spontaneous recovery profile of single-dose NMBAs [7] may be sufficient to ensure safety in this specific pediatric population and procedural context.

We acknowledge that objective monitoring remains the gold standard for research and for high-risk patients. However, our findings—supported by the pharmacokinetic/pharmacodynamic data from Vanlinthout et al. [7]—raise the hypothesis that, for low-risk children undergoing short, standardized procedures under sevoflurane maintenance, the incremental value of routine quantitative monitoring in predicting adverse events may be limited. Prospective studies incorporating objective monitoring are needed to validate this hypothesis and to define the optimal balance between monitoring-driven protocols and clinically driven pathways in ambulatory pediatric anesthesia.

### 4.7. Conclusions

This retrospective cohort study provides real-world data on the use of single-dose neuromuscular blocking agents (NMBAs) in children (ASA I-II, aged 3–12 years) undergoing brief otolaryngological procedures under sevoflurane-based anesthesia. The principal findings are:

(1) Dose-Response for Intubation Conditions: Rocuronium at 0.6 mg/kg (approximately 1.5 × ED₉₅) was associated with superior tracheal intubation conditions compared to 0.4 mg/kg rocuronium or 0.05 mg/kg cisatracurium (P < 0.01).
(2) Hemodynamic and Recovery Profiles: While the 0.6 mg/kg rocuronium group maintained higher mean arterial pressure at 1 and 5 minutes post-intubation (P < 0.05), extubation time, postoperative SpO₂, sedation levels, and emergence agitation scores were comparable across all groups.
(3) Safety Outcome: No clinical events attributable to residual neuromuscular blockade—hypoxemia (SpO₂ < 90%), unplanned reintubation, or severe emergence agitation—were observed in any of the 90 patients, despite the absence of routine pharmacological reversal or quantitative neuromuscular monitoring.

### 4.8. Interpretation and future directions

These real-world observations provide safety data supporting the exploration of simplified neuromuscular management strategies in this specific pediatric population. The absence of immediate adverse events suggests that, within this strictly defined context, a single 1–1.5 × ED₉₅ dose combined with rigorous clinical assessment may be sufficient to prevent clinically overt residual paralysis.

However, these findings should not be interpreted as evidence against quantitative neuromuscular monitoring, which remains strongly recommended by current guidelines [9,7,33–35] to minimize the risk of subclinical residual blockade and its associated pulmonary complications. Rather, they highlight an important question for future research: With quantitative monitoring (TOFr ≥ 0.9) as the mandatory safety baseline, can pharmacologically informed simplified management strategies optimize perioperative efficiency without increasing risk?

This question represents a critical next step toward the goal of pediatric ambulatory anesthesia that is both safe and efficient. Prospective, randomized trials incorporating quantitative monitoring are needed to determine whether, and in which populations, monitoring-driven protocols offer meaningful advantages over rigorous clinical assessment alone. Such studies would help define the optimal balance between safety and efficiency—moving beyond the question of “whether to monitor” to “how best to integrate monitoring into streamlined perioperative pathways.
